# Phenological stage and vegetation index for predicting corn yield under rainfed environments

**DOI:** 10.3389/fpls.2023.1168732

**Published:** 2023-07-21

**Authors:** Amrit Shrestha, Raju Bheemanahalli, Ardeshir Adeli, Sathishkumar Samiappan, Joby M. Prince Czarnecki, Cary Daniel McCraine, K. Raja Reddy, Robert Moorhead

**Affiliations:** ^1^ Department of Agricultural & Biological Engineering, Mississippi State University, Mississippi State, MS, United States; ^2^ Department of Plant and Soil Sciences, Mississippi State University, Mississippi State, MS, United States; ^3^ United States Department of Agriculture-Agricultural Research Service (USDA-ARS), Genetics and Sustainable Agriculture Research Unit, Mississippi State, MS, United States; ^4^ Geosystems Research Institute, Mississippi State University, Mississippi State, MS, United States

**Keywords:** corn, cover crop, phenology, remote sensing, vegetation indices, yield

## Abstract

Uncrewed aerial systems (UASs) provide high temporal and spatial resolution information for crop health monitoring and informed management decisions to improve yields. However, traditional in-season yield prediction methodologies are often inconsistent and inaccurate due to variations in soil types and environmental factors. This study aimed to identify the best phenological stage and vegetation index (VI) for estimating corn yield under rainfed conditions. Multispectral images were collected over three years (2020-2022) during the corn growing season and over fifty VIs were analyzed. In the three-year period, thirty-one VIs exhibited significant correlations (r ≥ 0.7) with yield. Sixteen VIs were significantly correlated with the yield at least for two years, and five VIs had a significant correlation with the yield for all three years. A strong correlation with yield was achieved by combining red, red edge, and near infrared-based indices. Further, combined correlation and random forest an alyses between yield and VIs led to the identification of consistent and highest predictive power VIs for corn yield prediction. Among them, leaf chlorophyll index, Medium Resolution Imaging Spectrometer (MERIS) terrestrial chlorophyll index and modified normalized difference at 705 were the most consistent predictors of corn yield when recorded around the reproductive stage (R1). This study demonstrated the dynamic nature of canopy reflectance and the importance of considering growth stages, and environmental conditions for accurate corn yield prediction.

## 1 Introduction

Feeding the growing population is an urgent challenge for the agriculture sector. However, over-fertilization to improve yield has resulted in negative consequences like water contamination and deterioration of soil health (Halliday and Wolfe, 1991; Singh, 2018). Fertilizer efficiency can be improved by applying the right amount at the right time based on the crop’s seasonal needs, and variable rate technologies (VRT) have that potential. For VRT, input decisions are usually based on a prescription map based on either intensive soil sampling (Kitchen et al., 2005; Servadio et al., 2017) or historic yield information (Lark, 1998; Jaynes et al., 2005). Spatially dense soil sampling (i.e., ≤ 2.5-acre grid which is the industry standard) is costly and not guaranteed to accurately represent soil conditions across space. At the same time, historic yield information is not reliable information for in-seasonal management decisions as yield varies from season to season (Maestrini and Basso, 2018). Therefore, in-season estimation of yield can improve management decisions for agricultural inputs.

It is estimated that 1.2 billion tons of corn was produced globally in 2021 (FAO, 2022) and the United States alone produced 382.9 million tons, worth of 82.6 billion USD (USDA, 2022). Corn is one of the important cereal crops, which is cultivated across the globe with the highest fertilization rate (IFA, 2022). The requirement for agricultural inputs (fertilizer and water) can be estimated by monitoring canopy optical properties. Green canopy has high absorption in the visible portion of the light spectrum and high reflectance in the near-infrared portion (Ustin and Jacquemoud, 2020). Plants absorb the maximum amount of light in the visible portion for photosynthesis where blue (B) and red (R) are strongly absorbed by the chlorophyll and carotenoid pigments (Lichtenthaler, 1987; Ustin and Jacquemoud, 2020), while, red edge (RE) and near-infrared (NIR) values are found to be associated with plant health (Horler et al., 1983; Fahrentrapp et al., 2019; Zahir et al., 2022). Additionally, leaf optical signatures are highly responsive to changes in soil and canopy nitrogen (Wood et al., 1992; Ziadi et al., 2008; Yang et al., 2012). Any detrimental events that change chlorophyll pigment reduce the potential assimilation capacity which adversely affects the growth, development and yield (Bheemanahalli et al., 2023). Consequently, changes in crop biochemical and physiological properties can be measured by monitoring canopy reflectance properties. The advancement of science and technologies has revolutionized high-throughput phenotyping, especially with UASs. The greatest boon has been its ability to collect high-resolution spatial and temporal information quickly, as well as flexibility on revisiting time and choice of sensor. Such high-resolution data can divulge the spatial and temporal variability (Matese et al., 2015) present in the crop during the growing season which can be integrated with VRT for management decisions. In addition, the adoption of VRT in combination with in-season spectral data for nitrogen application has promoted an increase in profitability and nitrogen saving as compared to traditional practices (Kitchen et al., 2010; Scharf et al., 2011).

A UAS comprises an uncrewed aerial vehicle (UAV), potentially one or more sensors or payloads, and a communicating and controlling device. The choice of sensor is an important consideration. A multispectral sensor that consists of blue, green (G), red, red edge, and near-infrared bands has been the first choice for studying crop bio-physiological parameters for many researchers (Yao et al., 2019; Bheemanahalli et al., 2022; Bheemanahalli et al., 2023). Various other spectral, thermal, and light detection and ranging (LiDAR) sensors have been deployed on UAV-based plant phenotyping (Xie and Yang, 2020). Both spectral and thermal sensors have been used for studying plant health (Geipel et al., 2014; Simic Milas et al., 2018) and LiDAR data has been used for plant structure (Yuan et al., 2018; Wu et al., 2019; ten Harkel et al., 2020; Luo et al., 2021). Santana et al. (2021) deployed a four-band multispectral sensor mounted on a UAV for the estimation of corn yield. Vong et al. (2021) used a red-blue-green (RGB) sensor to estimate corn stand count. Barzin et al. (2020) used a five-band multispectral sensor (blue, green, red, RE, NIR) for the estimation of corn yield. Chivasa et al. (2021) used a four-band multispectral sensor (green, red, RE, NIR) for the prediction of corn streak virus severity and yield. Zhang et al. (2019) used hyperspectral imagery collected from a UAV for detecting yellow rust disease in wheat. Crusiol et al. (2020) used a thermal sensor mounted on a UAV for determining water status of soybean plants.

The sensor is mounted on a UAV and deployed to collect the imagery data. These data are generally transformed into a ratio or combination of normalized differences of spectral bands known as a vegetation index (VI) to study crop health and performance. Vegetation indices (VIs) have been found highly related to physiological parameters (Bannari et al., 1995; Xue and Su, 2017; Ma et al., 2019) and less sensitive to atmospheric effects compared to spectral bands (Myneni and Asrar, 1994; Gitelson et al., 2002). The normalized difference vegetation index (NDVI) is commonly used to measure vegetation health. It exhibits a strong association with leaf dry biomass (Kross et al., 2015), yield (Hassan et al., 2019; Maresma et al., 2020), and leaf area index (LAI) (Shafian et al., 2018). However, its usefulness has been limited due to saturating tendency at the higher LAI (Sellers, 1985; Carlson and Ripley, 1997). Various other VIs are found to have a strong association with crop bio-physiological parameters: normalized difference spectral index (NDSI) and soil adjusted vegetation indices (SAVI) are found to have a strong correlation with leaf nutrition index (Zhao et al., 2018), modified normalized difference water index (MNDWI) with LAI (Zarate-Valdez et al., 2012), MERIS terrestrial chlorophyll index (MTCI) with chlorophyll (Dash and Curran, 2007), wide dynamic range vegetation index (WDRVI) with yield (Maresma et al., 2016), and crop water stress index (CWSI) with water status (Gonzalez-Dugo et al., 2014; Santesteban et al., 2017). In addition, different techniques were used to select vegetation indices based on variance inflation factor, recursive feature elimination, random forest, correlation, Bayesian variable selection, and genetic algorithm for yield estimation (Maya Gopal and Bhargavi, 2019; Barzin et al., 2020; Aditya Shastry and Sanjay, 2021; Saravi et al., 2021; Barzin et al., 2022). A random forest was widely used to select VIs to estimate different crop traits (Maya Gopal and Bhargavi, 2019; Barzin et al., 2020; Li et al., 2022; Luo et al., 2022) due to its robustness to outliers and noise (Breiman, 2001).

To our best knowledge, none of the studies have reported suitable VIs that can be used throughout the growing season to predict yield in corn. Thus, the objectives of our research were to i) identify the potential VIs that significantly correlate with yield across the growing season, ii) identify the suitable phenological stage for yield prediction and iii) identify the best predictive VI for yield estimation under rainfed environments.

## 2 Materials and methods

### 2.1 Site description

**Figure 1:**
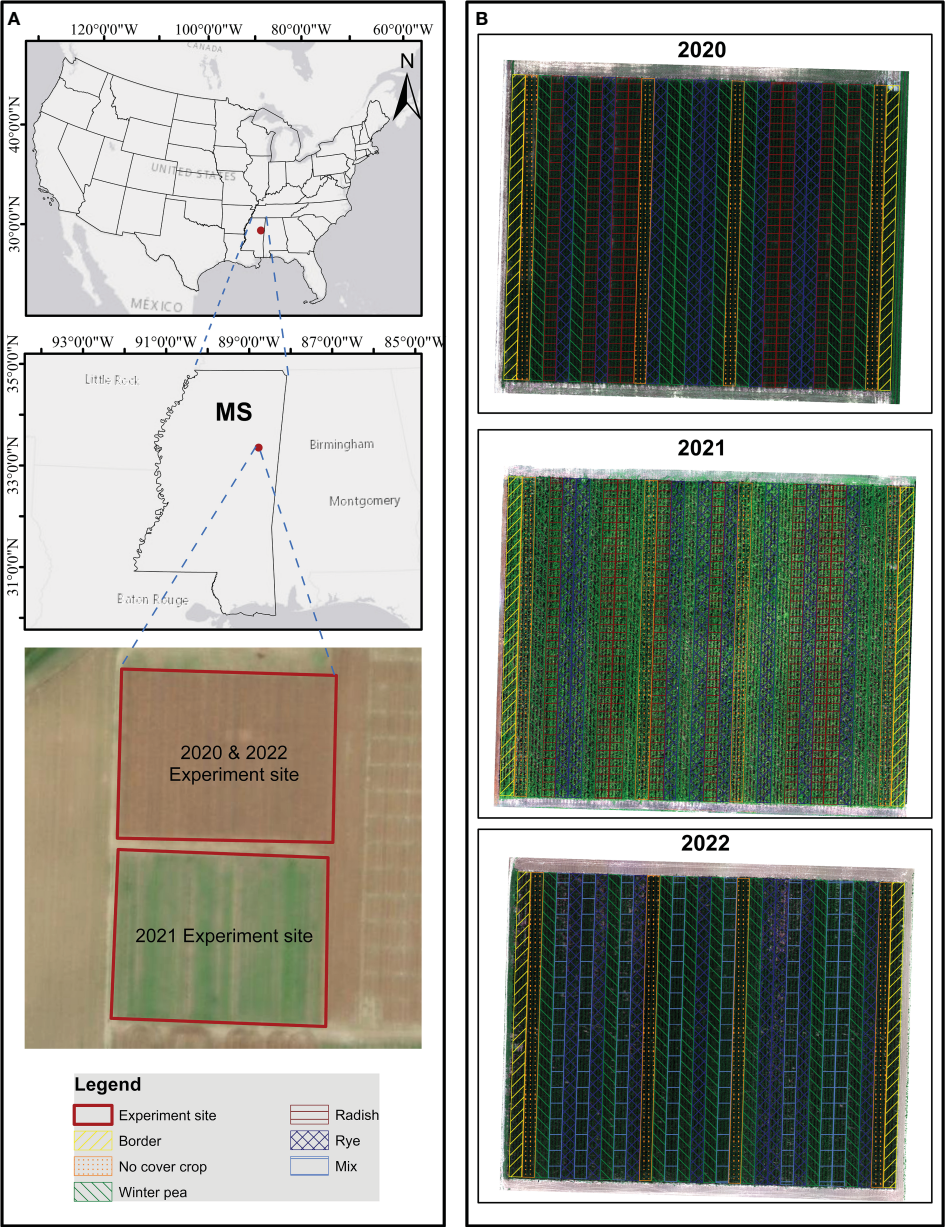
Research site location **(A)** and experiment design **(B)**.

The field experiment was conducted for three consecutive years 2019-2022 in the R. R. Foil Plant Science Research Center, Mississippi State University (33°28’21.0”N 88°46’25.5”W), **Figure 1**.

### 2.2 Experimental design and crop husbandry

Corn - and - cotton was grown as a rotation cash crop during the growing season under rainfed conditions. Best management practices were adopted to establish the crops: cover crops were grown during the fallow period with minimum or no tillage and cash crop was planted in mid-April. The experiment consisted of three cover crop treatments, Austrian winter pea (*Pisum sativum* L.), Daikon radish (*Raphanus sativus* L. subsp. *longipinnatus*), cereal rye (*Secale cereale* L. var. Elbon), and a no cover crop (NCC) treatment. In 2022, radish was replaced with a mixture of winter pea, radish, and rye. The experiment was designed as a split-plot randomized complete block, with the cash crop as the main factor and the cover crop species as the sub-factor. Each subplot had four rows of 90 m length × 3.8 m width, with four replicates (**Figure 1**). Cover crops were planted in October and terminated in March across three years. Prior to the cover crop planting, two tons per acre of poultry litter was surface broadcasted. After the termination of the cover crop, corn was planted on April 14, 2020, April 07, 2021, and March 28, 2022. A split fertilization procedure was adopted and divided into two parts. No cover crop (NCC) treatment received 56 kg N ha^-1^ as a starter fertilizer at the V3 and 168 kg N ha^-1^ at the V12 growth stage. The cover crop treatments did not receive starter fertilizer, but 168 kg N ha^-1^ was applied to cover crop treatments at the V12 growth stage. The source of N fertilizer was urea ammonium nitrate (UAN solution, 32% N). Corn plots were harvested in September 2020-2022 after completing physiological maturity. It is important to note that, in this study, the cover crop treatment’s influence on corn yield was not the major focus. Instead, the study aims to determine the ideal phenological stages and likely highest predictive power VIs for corn yield prediction under rainfed conditions.

### 2.3 Data collection

#### 2.3.1 Yield

The middle two rows of each subplot were harvested using a mechanical corn harvester and the yields were adjusted to a 15.5% moisture level. The obtained yield was then converted into the mega-gram per hectare (Mg ha^-1^).

#### 2.3.2 Data acquisition

A UAV mounted with a five-band multispectral camera (Rededge MX, Micasense Inc., Seattle, USA) was flown at an altitude of 61 m (200 ft) above ground level producing a spatial resolution of approximately 4 cm. The sensor has the following central wavelengths(bandwidths): 475(20) nm, 560(20) nm, 668(10) nm, 717(10) nm, and 842(40) nm for blue, green, red, RE, and NIR bands, respectively. A mission planning software (DJI GSPro, DJI LLC., Shenzen, China) was used to create the UAV flight plan in a single grid pattern. Images were acquired by setting the sensor in automatic exposure mode with 80% frontal overlapping and 70% side overlapping. The data was stored in 16-bit raw format. Before and after each flight, an image of the reference reflectance calibration tile provided by the sensor manufacturer was taken, which was later used for reflectance calibration following guidance from the sensor manufacturer. The data were collected weekly throughout the year as the weather permitted within ±2 hours of local solar noon. The UAV data were collected between the vegetative stage (V3) and the reproductive stage (R5) as shown in **Supplementary Table S1**. Specifically, data were collected at various time points, including V5, V6, V7, V10, V11, and V13, which represent the number of visible leaf collars on the main stem, where Vn refers to leaf collars greater than 13. In addition, data were collected at the reproductive stage, including R1, R2, R3, R4, and R5, which correspond to the silk, blister, milk, dough, and dent stages, respectively (Nleya et al., 2016).

### 2.4 Data preprocessing

A commercial image stitching software Pix4D Mapper (Pix4D SA, Lausanne, Switzerland) was used to generate a single orthomosaic image of the whole field. The mosaic image was geo-rectified using the ground control points that were placed around the edge of the experimental field. The accuracy of the GPS device (Trimble Geo7x, Trimble Inc., CA, USA) was ±2cm. The image digital number was converted into reflectance value using the reference reflectance calibration tile provided with the sensor. To extract the corn pixels, a support vector machine algorithm was employed using geospatial software (ENVI, version 5.6, Exelis Visual Information Solution, Boulder, CO, USA). The orthomosaic image can be classified into three major classes: corn, soil and weed pixels. For each class, at least five thousand pixels were selected for classification. The accuracy for the classification of corn pixels for all the images was greater than 0.95.

### 2.5 VI extraction and selection

Orthomosaic images that were affected by clouds were removed from the analysis. After removing the background pixels (i.e., soil, stubble and weeds) from orthomosaic, the middle two rows of each subplot were first digitized and split into individual subplots using ArcGIS (Environmental Systems Research Institute, Inc. (ESRI), Redland, CA, USA). Each subplot was loaded in Python using the GDAL library to compute the average spectral information. This spectral information was then used to calculate the VIs that are related to crop health, photosynthesis, biochemical, and physiology. The list of VIs used in this study is given in **Supplementary Table S2**. Correlation analysis between VIs and yield was performed for each flight or during the corn growing season. Further, to select the VI with the best predictive power we followed three criteria: i) any VI that had a significant correlation (absolute value of correlation coefficient r ≥ 0.7) with yield for each date was assigned a score of 1, ii) only those VIs that scored 1 were summarized in each year, and iii) the top three VIs with the maximum cumulative score were identified as stable across stages of growth as well as the VIs with the best predictive power. We repeated the same analysis for three growing seasons and selected the VIs that were unique and common across years as the best predictors of yield. In previous studies (Asuero et al., 2006; Akoglu, 2018; Kogan et al., 2018), a correlation coefficient with an absolute value greater than 0.7 has been widely regarded as a strong correlation. Therefore, we chose a threshold limit of 0.7 to assign a score of 1.

In addition, to determine the best VI at a given phenology stage, a machine learning technique based on random forest (RF) algorithm was implemented. A RF is collection of decision trees that are trained independently. The prediction from each decision tree is averaged to a single output (Breiman, 2001). Initially, the RF model parameters were tuned by implementing a randomized search cross-validation method. In this method, a set of predefined values were randomly chosen as a possible candidate, and a hundred sets of hyperparameters were considered. Next, cross-validation was performed for each set of hyperparameters, and five-fold cross-validation was chosen. Then finally, the model returns the best set of hyperparameters. The tuned hyperparameters were the number of trees, number of samples required to split the internal node, minimum samples required to be a leaf node, and maximum feature to be considered while splitting the node (full feature and square root of total number of feature). A bootstrap technique was selected for generating tree samples. After tuning the RF model, the model was fitted with the best set of hyperparameters. To rank the variables, a permutation-based feature importance method was implemented utilizing all the variables, and a simple linear model was developed with the top-ranked variable. Geospatial software, ArcGIS and ENVI were used for data manipulation. The modeling was done with Python (version 3.9) using the stats models (version 0.13.5) library for linear regression analysis and the sci-kit-learn library (version 1.0.2) for random forest analysis.

### 2.6 Yield modeling

A simple linear regression model was employed to develop a linear relationship between corn yield and selected variables as discussed above. The model performance was compared with the coefficient of determination (R^2^) and mean absolute percentage error (MAPE). The variable with high R^2^ with significantly lower MAPE (*p*<0.01) was considered the highest predictive power VI across the growing season. The modeling was done with Python (version 3.9) using the sci-kit-learn library (version 1.0.2).

## 3 Results

**Figure 2:**
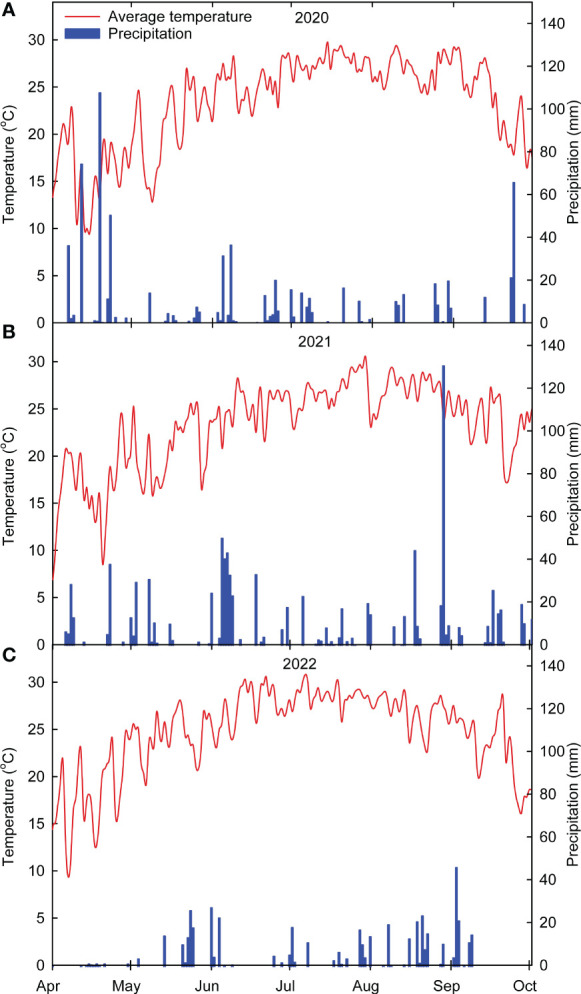
Observed daily air temperature and precipitation variability during the experimental period (2020 – **(A)**, 2021 – **(B)** and 2022 – **(C)**. Weather data were obtained from the Delta Agricultural Weather Center (http://deltaweather.extension.msstate.edu/) for the experiment site.

**Figure 3:**
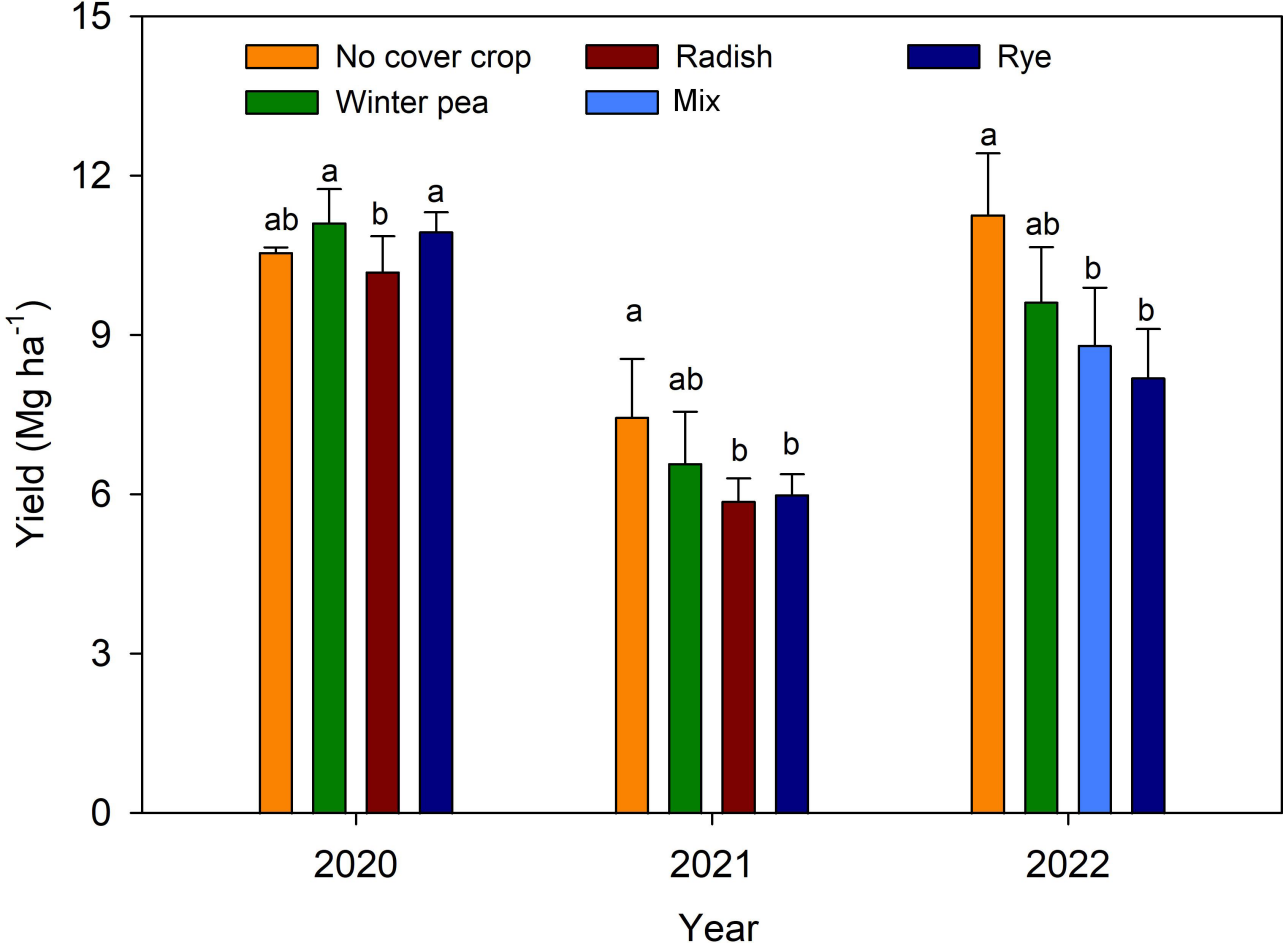
Corn yield variability in response to cover crop treatments. Vertical bars denote mean ± standard deviation (SD). Bars with common letters are not statistically significant at *p<0.05* (LSD test) within a year.

During the corn growing season in 2020, 2021 and 2022, the observed average air temperature was 24.2, 23.7, and 24.7°C, respectively, and precipitation was 523 mm (optimum), 859 mm (high) and 481 mm (low), respectively (**Figure 2**). The year 2022 was observed to be relatively drier than 2020 and 2021. There were significant differences in corn yield between treatments and years (**Figure 3**). Plots with winter pea and cereal rye treatments recorded higher yields than radish treatment in 2020. Cover cropping treatments (radish or mix and rye) yielded significantly lower than control plots in 2021 and 2022 except winter peas. The growing season with optimum rainfall (2020) was associated with a higher yield, while maximum rainfall (2021) during the growing season was associated with a lower yield. The annual mean yield for 2020, 2021 and 2022 were 10.72, 6.34 and 9.22 Mg ha^-1^, respectively (**Figure 3**).

### 3.1 Correlation analysis

**Figure 4:**
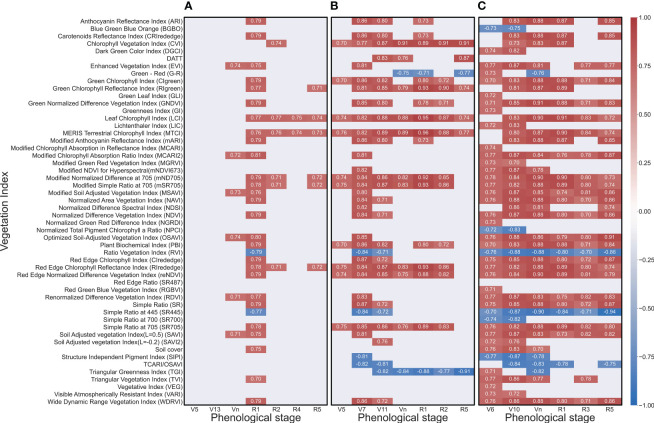
Heat map showing the significant correlation coefficient (r≥0.7) between corn yield and vegetation index at different growth stages in 2020 **(A)**, 2021 **(B)** and 2022 **(C)**. Darker color signifies a high correlation, while lighter color signifies a weaker correlation between yield and corresponding vegetation index.

Results indicated a set of VIs (12 in 2020 and 2021, and 28 in 2022) with correlation coefficients |r| ≥ 0.7 at least twice in the same growing season (**Figure 4**, **Supplementary Table S3**). In 2020, MTCI and leaf chlorophyll index (LCI) had the strongest positive correlation with the yield at four timings (R1, R2, R3, and R5 growth stages) and were ranked first. The modified normalized difference at 705 (mND705), modified simple ratio at 705 (mSR705), and red edge chlorophyll reflectance index (RIrededge) had significant correlations with corn yield at three timings. While seven VIs such as enhanced vegetation index (EVI), modified chlorophyll absorption ratio index (MCARI2), soil adjusted vegetation index (SAVI), modified SAVI (MSAVI), optimized SAVI (OSAVI), renormalized difference vegetation index (RDVI), and green chlorophyll reflectance index (RIgreen) had significant correlations with yield at only two timings. The MCARI2 showed the highest correlation with yield at R1 growth stage (r=0.81) (**Figure 4**). In 2021, CVI, LCI and MTCI had strongest correlations across all (V5, V7, V11, Vn, R1, R2, and R5) growth stages (first tier). The second tier was dominated by RE-based VIs, while third tier includes green chlorophyll index (CIgreen) and triangular greenness index (TGI) (**Figure 4**). A strong negative correlation (r=-0.91) was noted between TGI and the yield at R5 (119 DAP). At R1 (83 DAP), the highest correlation (r=0.96) between yield and MTCI was observed. In 2022, there were 20 VIs that fall under top tier. This group was dominated with RE-based indices and showed a strong correlation with yield across the six (V6, V10, V19, V1, V3 and V5) growth stages. The second tier VIs such as EVI, LCI and MTCI showed significant correlations with yield for five times. The third-tier VIs were TCARI/OSAVI, TVI, ARI CRIrededge and mARI. Further, green normalized difference vegetation index and leaf chlorophyll index showed strong positive correlations (r=0.91) with yield at Vn (67 DAP) and R1 (78 DAP), respectively. At R5 (108 DAP), a significant negative correlation (r=-0.95) was found between SR445 and yield (**Figure 4**).

### 3.2 Unique and common VIs across the year for yield prediction

**Figure 5:**
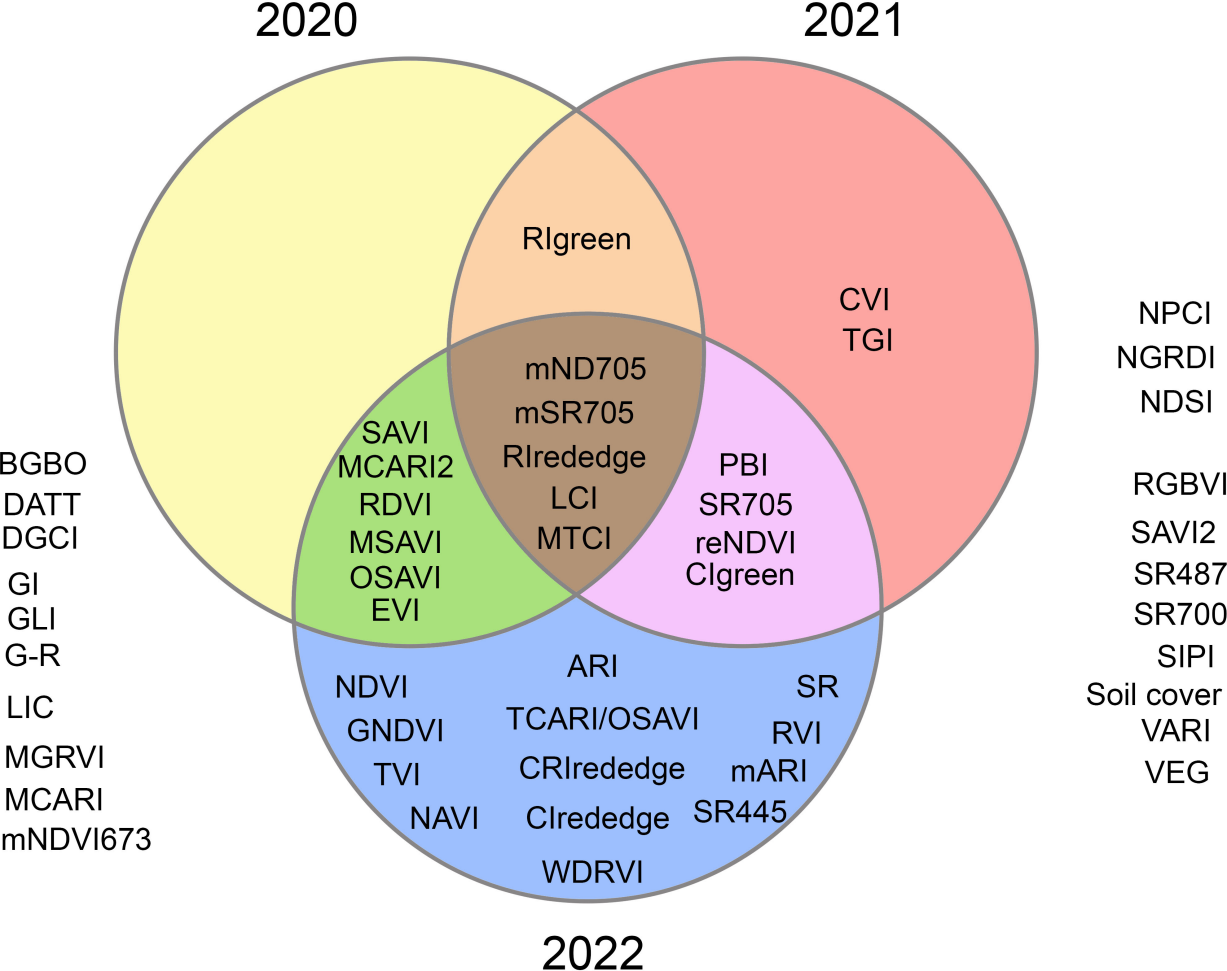
A Venn diagram showing the vegetation indices (VIs) with the highest predictive power within (unique VIs) or between years (common VIs). Full form of acronyms is given in **Figure 4** and the formulation are given in **Supplementary Table 2**.

**Figure 6:**
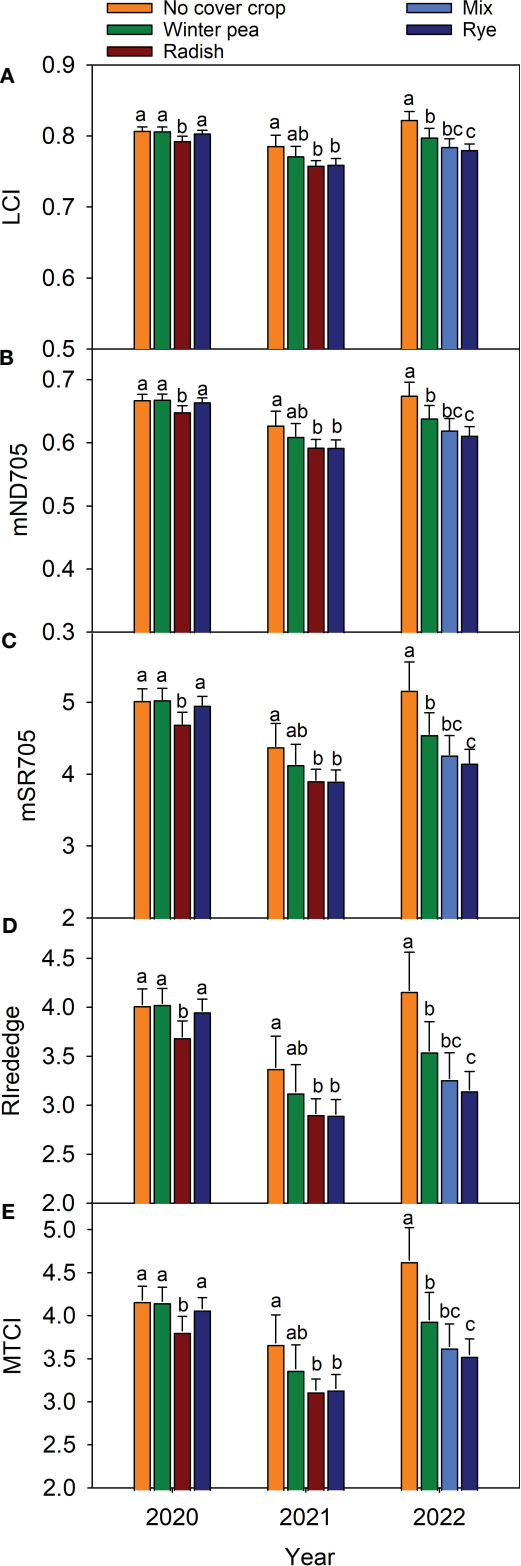
Influence of cover crop on the spectral properties of corn at R1 growth stage. Leaf Chlorophyll Index (LCI, **A**), Modified Normalized Difference at 705 (mND705, **B**), Modified Simple Ratio at 705 (mSR705, **C**), Red Edge Chlorophyll Reflectance Index (RIrededge, **D**), and MERIS Terrestrial Chlorophyll Index (MTCI, **E**). Vertical bars denote mean ± SD. Treatments with common letters are not statistically significant at *p<0.05* (LSD test) within a year.

Across all three years, the five VIs (mND705, mSR705, RIrededge, LCI, and MTCI) with the highest predictive power were identified (**Figure 5**). In 2021, two unique VIs were identified, while thirteen were noted in 2022. Six VIs (EVI, MCARI2, MSAVI, OSAVI, RDVI and SAVI) were unique to 2020 and 2022; CIgreen, PBI, reNDVI and SR705 were unique to 2021 and 2022. RIgreen was common in both 2020 and 2021 (**Figure 5**). Five VIs common to all growing seasons were used to build linear regression models for each growing season (**Table 1**). The results of the models revealed improvement in yield estimation as the season progressed with the best prediction at the R1 stage: with mND705 (R^2 ^= 0.62) in 2020, with MTCI (R^2 ^= 0.92) in 2021, and with LCI (R^2 ^= 0.82) in 2022. Thereafter, the performance became relatively weaker (**Table 1**). The R^2^ and mean absolute percentage error (MAPE) of various models ranged from 0.04 to 0.92 and 3.01% to 11.85%, respectively (**Table 1**). A higher error was generally noted at the early or late growth stages. Although the association was weaker, RIrededge, mSR705 and mND705 performed better than LCI and MTCI at the early growth stage. However, mND705 had higher predictive power throughout the growing season compared to RIredege and mSR705 (**Table 1**). In addition, MTCI had 8 times the highest performance (high R^2^ with low MAPE), while LCI and mND705 had the highest performance for six times. All common VIs that are sensitive to changes in chlorophyll content had the ability to separate cover crop treatments at the R1 growth stage across years (**Figure 6**). In general, higher values of VIs at the R1 stage were associated with higher yields and vice versa. In 2021, all VIs followed a similar trend of yield response to cover crop treatment. At the reproductive stage, RIrededge, mSR705 and MTCI had a higher ability to differentiate cover crop treatments (winter pea and cereal rye) from the control (no cover crop) under rainfed conditions (**Figure 6**). Lower MTCI values in radish or mix and rye treatments at R1 were associated with lower yields (**Figure 6E**).

**Table 1:** Association between stable VIs collected at different phenological stages and corn yield under rainfed environments.

Growth stage	LCI		MTCI		RIrededge		mSR705		mND705	
	R^2^	MAPE	R^2^	MAPE	R^2^	MAPE	R^2^	MAPE	R^2^	MAPE
2020										
V5	0.04ns	4.65	0.04ns	4.65	0.18*	**4.07**	0.18*	**4.07**	0.18*	**4.07**
V13	0.24**	**4.26**	0.21*	4.36	0.17**	4.46	0.17*	4.46	0.19*	4.39
Vn	0.47***	3.44	0.43***	3.58	0.44***	3.57	0.44***	3.57	0.48***	**3.42**
R1	0.59***	3.07	0.58***	3.12	0.60***	3.03	0.60***	3.03	0.62***	**2.99**
R2	0.59***	**3.12**	0.58***	3.18	0.51***	3.42	0.51***	3.42	0.51***	3.4
R4	0.56***	**3.18**	0.55***	3.23	0.46***	3.54	0.46***	3.54	0.47***	3.51
R5	0.55***	**2.97**	0.54***	3.01	0.51***	3.17	0.51***	3.17	0.52***	3.17
2021										
V5	0.55***	7.05	0.58***	**6.85**	0.57***	7.02	0.57***	7.02	0.55***	7.13
V7	0.68***	5.64	0.68***	5.73	0.70***	5.5	0.70***	5.5	0.70***	**5.45**
V11	0.77***	5.16	0.79***	**4.89**	0.76***	5.31	0.76***	5.31	0.74***	5.49
Vn	0.78***	5.14	0.80***	**4.81**	0.69***	5.92	0.69***	5.92	0.67***	6.15
R1	0.90***	3.51	0.92***	**3.12**	0.86***	3.87	0.86***	3.87	0.84***	4.16
R2	0.76***	4.81	0.77***	**4.72**	0.74***	5.12	0.74***	5.12	0.72***	5.35
R5	0.55***	7.42	0.59***	**7.0**	0.49***	7.49	0.49***	7.49	0.46***	7.72
2022										
V6	0.09ns	11.85	0.09ns	11.85	0.60***	7.8	0.60***	7.8	0.60***	**7.71**
V10	0.69***	7.39	0.65***	7.72	0.67***	7.44	0.67***	7.44	0.71***	**7.11**
Vn	0.81***	**5.51**	0.76***	6.29	0.77***	6.18	0.77***	6.18	0.81***	5.53
R1	0.82***	**5.79**	0.81***	5.89	0.79***	6.16	0.79***	6.16	0.80***	6.09
R3	0.70***	7.13	0.70***	**7.06**	0.64***	7.88	0.64***	7.88	0.63***	8.05
R5	0.53***	8.68	0.54***	**8.55**	0.54***	8.65	0.54***	8.65	0.53***	8.76

Leaf Chlorophyll Index (LCI), MERIS Terrestrial Chlorophyll Index (MTCI), Red Edge Chlorophyll Reflectance Index (RIrededge), Modified Simple Ratio at 705 (mSR705) and Modified Normalized Difference at 705 (mND705). *, ** and *** indicate the regression is significant at *p*<0.05, *p*<0.01 and *p*<0.001 respectively. ‘ns’ indicates non-significant. MAPE: mean absolute percentage error, DAP: day after planting. Bold values represent the best VI for a given growth stage.

### 3.3 Variable assessment

A RF machine learning algorithm was implemented to identify the VIs with the highest predictive power (best) at different growth stages (**Table 2**). The R^2^ and MAPE of the various model considered with the RF method ranged from 0.25-0.89 and 2.82-9.20%, respectively (**Table 2**). In 2020, the highest predictive power of VIs at V5 and V13 were the visible atmospherically resistant index (VARI), while at Vn, R1, R2, R4, and R5 were visible MSAVI, soil cover, triangular vegetation index (TGI), LCI, and MTCI, respectively. In 2021, the most predictive power VIs were MTCI (V5), RVI (V7), DATT (V11), CVI (Vn and R1), LCI (R2), and TGI (R5). However, in 2022, the VIs with the highest predictive power were RIgreen, TCARI/OSAVI, SR445, CVI, SAVI, and SR445, corresponding to growth stages V6, V10, Vn, R1, R3, and R5. Particularly, the reproductive stage exhibited the strongest prediction power for these VIs. At the R1 stage, the best variables ranked by the RF method had a strong association with yield (**Table 2**): soil cover (R^2 ^= 0.56) in 2020, CVI (R^2 ^= 0.82) in 2021, and CVI (R^2 ^= 0.76) in 2022. However, at the same growth stage, the VIs selected from correlation analysis had higher predictive power in estimating yield: (mND705, R^2 ^= 0.62) in 2020, (MTCI, R^2 ^= 0.80) in 2021, and (LCI and mND705, R^2 ^= 0.81) in 2022 (**Table 1**).

**Table 2:** A simple linear regression model statistic developed with the top-ranked vegetation index selected by a random forest based variable selection method for three years of study.

	2020			2021				2022			
Growth stage	VI	R^2^	MAPE	Growth stage	VI	R^2^	MAPE	Growth stage	VI	R^2^	MAPE
V5	VARI	0.25**	4.25	V5	MTCI	0.58***	6.85	V6	RIgreen	0.47***	9.2
V13	VARI	0.39***	3.63	V7	RVI	0.70***	6.19	V10	TCARI/OSAVI	0.71***	7.19
Vn	MSAVI	0.53***	3.21	V11	DATT	0.69***	5.83	Vn	SR445	0.80***	5.74
R1	Soil cover	0.56***	2.82	Vn	CVI	0.82***	4.04	R1	CVI	0.76***	6
R2	TGI	0.43***	3.61	R1	CVI	0.79***	4.54	R3	SAVI	0.67***	7.69
R4	LCI	0.56***	3.18	R2	LCI	0.76***	4.81	R5	SR445	0.89***	4.21
R5	MTCI	0.54***	3.01	R5	TGI	0.83***	4.59				

V5, V6, V7, V10, V11, and V13, represent the vegetative growth stage with 5, 6, 7, 10, 11 and 13 visible leaf collars on the main stem. Vn represent number of leaf collars greater than 13. R1, R2, R3, R4, and R5, which correspond to the silk, blister, milk, dough, and dent stages, respectively. *, ** and *** indicate the regression is significant at *p*<0.05, *p*<0.01 and *p*<0.001 respectively. ‘ns’ indicates non-significant. VI- vegetation index; MAPE: mean absolute percentage error. Full form of acronyms are given in **Figure 4** or **Supplementary Table 2**.

## 4 Discussion

Crop yield is a complex result of genetics, environmental factors, and management practices. Researchers aim to improve yield through strategic selection of genotypes/hybrids, while farmers focus on optimizing inputs. In both scenarios, the common goal is to maximize crop yield, which is viewed as important because of its association with economic value. Studies, including the one presented herein, show significant variability in yield from year to year, with greater variability within a year rather than between years (Dhillon et al., 2022). This variability is primarily due to environmental factors such as soil (Bresler et al., 1981) and rainfall (Or and Hanks, 1992). To complement the ongoing season-based studies to make more informed management decisions, this study identified suitable growth stages and VI/s for predicting corn yield. The intention is that such a method can serve as an alternative and improvement to relying solely on historical data.

Mapping yield early help diagnose plant health or yield variability and create management zones for in-season decision making. For example, with this knowledge, a farmer can allocate sampling resources to regions of interest, reducing overall cost while improving knowledge gained from the activity. With corn being heavily reliant on fertilization, the yield map can serve as a prescription map for VRT for agricultural input management. A UAS can collect information at high resolution in contrast to satellite remote sensing, which can be beneficial for finer-scale management. Nevertheless, the extraction of information from imagery and the selection of VI have a significant effect on yield prediction or mapping. This study used a correlation-based feature selection method and compared it to a random forest approach. Results indicated that different variables are important at different growth stages and can vary by year (**Table 1**, **2**), as seen in previous studies (Barzin et al., 2020). In addition, the results of feature selection using correlation analysis demonstrated an ability to make early predictions, compared to the RF approach.

This three-year study indicated that the VIs’ ability to predict yields is weaker at the early vegetative stage compared to the reproductive stage due to slower growth and canopy. This study identified five VIs (LCI, MTCI, mND705, mSR705, and RIrededge) that had a significant correlation with yield across years (**Figure 5**). The commonality in these VIs are NIR and RE bands. Although reNDVI is based on NIR and RE, this VI was not selected due to a weaker correlation in 2020 (**Figure 4**). However, this VI has been correlated with canopy chlorophyll and LAI around the reproductive stage in corn (Simic Milas et al., 2018). In addition, Li et al. (2014) and Zhao et al. (2007) reported that three-band indices were better estimators of plant nitrogen concentration and uptake compared, and LAI and above-ground biomass, respectively, compared to two-band indices. In this study, LCI, MTCI and mND705 were found to be superior compared to mSR705 and RIrededge in yield estimation at the R1 stage (**Table 1**).

Chlorophyll content in leaves reaches its peak during the R1-R3 stages and is a vital pigment for photosynthesis (Schepers et al., 1992; Brewer et al., 2022). Thus, LCI (Datt, 1999), MTCI (Dash and Curran, 2004) and mND705 (Sims and Gamon, 2002) have been used as proxies for canopy greenness or chlorophyll content. High correlations were reported for LCI with chlorophyll *a* (r = 0.86), and chlorophyll *a+b* (r=0.84) (Datt, 1999). Similarly, MTCI had strong relationships with chlorophyll content (Dash et al., 2010), photosynthesis (Maleki et al., 2020), nitrogen uptake (Li et al., 2021) and yield (Zhang and Liu, 2014). mND705 has also been utilized to track the senescence dynamics of wheat accessions (Anderegg et al., 2020). The blue reflectance signal from crops is a combination of chlorophyll and carotenoid pigments, while the red reflectance is mainly dominated by chlorophyll. The prevalence of chlorophyll and photosynthesis information in MTCI likely led to better prediction results when combined with the RE and NIR bands, compared to the combination of blue, RE, and NIR in mND705, mSR705, and RIrededge. Further, the prediction ability of MTCI were comparable or better than the prediction ability of the preferred VIs by the RF algorithm.

We investigated the use of various VIs as indicators of canopy greenness or photosynthetic pigments to examine crop health (**Figure 6**) and yield (**Figure 3**) responses to management. Identified five high predictive VIs (LCI, mND705, mSR705 RIrededge and MTCI) found to be linked to the plant’s ability to capture and use light energy for growth and development (Boyd et al., 2011; Dong et al., 2015; Barnes et al., 2017; Tan et al., 2018). Moreover, these VIs are also sensitive to changes in chlorophyll content and canopy structure (Sims and Gamon, 2002; Wang et al., 2017; Croft et al., 2020). For instance, a higher LCI value indicates greater photosynthetic efficiency and nitrogen content in corn, which is often associated with higher yields. On the other hand, lower MTCI values under radish or mix and rye treatments at R1 indicate lower chlorophyll content and poor canopy structure, thus lower yields compared to other treatments (**Figure 6**). Similarly, higher values of other VIs were also associated with increased chlorophyll content and greater yields across years. It is evident that VIs that are sensitive to changes in pigments can assist in mapping differences in plant health and yield potential in corn in response to cover cropping systems under rainfed conditions (**Supplementary Table 4**). Identified five promising VIs can help monitor plant health and yield potential, which can be used to guide effective crop management practices, such as fertilization, irrigation, and pest management.

In summary, a combination of either blue or red, RE, and NIR-based vegetation indices had a strong correlation with corn yield. Our results showed that certain vegetation indices demonstrated high consistency in predicting corn yield. Specifically, the indices LCI, MTCI, mND705, mSR705, and RIrededge showed the strongest predictive capabilities. Among them, MTCI emerged as the most promising VI and can be effectively used to predict corn yield during the reproductive stage. Further, weekly cloud-free imagery can be used for real-time monitoring of yield estimation under different cropping systems, which can support both research and farm decision-making as UASs become increasingly ubiquitous.

## Data availability statement

The original contributions presented in the study are included in the article/**Supplementary Material**. Further inquiries can be directed to the corresponding author.

## Author contributions

RB, SS, and JC conceptualized the research; RM provided the funding. AS, CM, and AA collected the data. AS and RB performed the image analysis, data analysis, data visualization, and composed the first draft. All authors contributed to editing and revision. All authors contributed to the article and approved the submitted version.
