# Supplementary material for: Phenological stage and vegetation index for predicting corn yield under rainfed environments
Source: Front Plant Sci. 2023 Jul 21;14:1168732. doi: 10.3389/fpls.2023.1168732 (PMC10401276; doi:10.3389/fpls.2023.1168732)
Supplement: Supplementary file 1 [file DataSheet_1.zip › Supplementary Tables.DOCX]

Supplementary Material

Phenological Stage and Vegetation Index for Predicting Corn Yield under Rainfed Environments

Amrit Shrestha^1^, Raju Bheemanahalli^2*^, Adeli Ardeshir^3^, Sathishkumar Samiappan^4^, Joby M. Prince Czarnecki^4^, Cary Daniel McCraine^4^, K. Raja Reddy^2^, Robert Moorhead^4^

***Correspondence:**

Dr. Raju Bheemanahalli, Plant Stress Physiology, Email: [rajubr@pss.msstate.edu](mailto:rajubr@pss.msstate.edu)

# Supplementary Tables

# Supplementary Table 1. Summary of UAV data collection during the growing seasons along with associated growth stages.

| **2020** | | | **2021** | | | **2022** | | |
| --- | --- | --- | --- | --- | --- | --- | --- | --- |
| **Growth stage** | **DAP*** | **GDD**** | **Growth stage** | **DAP*** | **GDD**** | **Growth stage** | **DAP*** | **GDD**** |
| V5 | 36 | 297 | V5 | 37 | 298 | V6 | 38 | 327 |
| V13 | 56 | 574 | V7 | 44 | 378 | V10 | 50 | 487 |
| Vn | 71 | 791 | V11 | 55 | 515 | Vn | 67 | 741 |
| R1 | 77 | 891 | Vn | 72 | 766 | R1 | 78 | 920 |
| R2 | 91 | 1132 | R1 | 83 | 935 | R3 | 93 | 1199 |
| R4 | 100 | 1299 | R2 | 96 | 1141 | R5 | 108 | 1476 |
| R5 | 107 | 1422 | R5 | 119 | 1536 |  |  |  |

Note: V5, V6, V7, V10, V11, and V13, represent the number of visible leaf collars in the main stem, where Vn refers to leaf collars greater than 13. In addition, data were collected at the reproductive stage, including R1, R2, R3, R4, and R5, which correspond to the silk, blister, milk, dough, and dent stages, respectively. A base temperature of 10 °C is used for calculating growing degree days.

*DAP: days after planting corresponds to UAV data collection

**GGD: Growing degree days

# Supplementary Table 2. List of vegetation indices used in this study.

| **Vegetation Index** | **Category** | **Reference** |
| --- | --- | --- |
| Anthocyanin Reflectance Index (ARI) | Anthocyanins | (Gitelson and Merzlyak, 2004) |
| Blue Green Blue Orange (BGBO) | Lutein zeaxanthins | (Fyfe, 2003) |
| Carotenoids Reflectance Index (CRIrededge) | Carotenoids | (Gitelson and Merzlyak, 2004) |
| Chlorophyll Vegetation Index (CVI) | Chlorophyll | (Vincini et al., 2008) |
| Dark Green Color Index (DGCI) | Greenness | (Karcher and Richardson, 2003; Rorie et al., 2011) |
| DATT | Chlorophyll | (Datt, 1998) |
| Enhanced Vegetation Index (EVI) | Green Bio estimation | (Huete et al., 2002) |
| Green – Red (G-R) | Ground cover, biomass | (Prabhakara et al., 2015) |
| Green Chlorophyll Index (CIgreen) | Chlorophyll | (Gitelson et al., 2005) |
| Green Chlorophyll Reflectance Index (RIgreen) | Chlorophyll | (Gitelson, 2004; Costa et al., 2021) |
| Green Leaf Index (GLI) | Crop cover | (Louhaichi et al., 2001) |
| Green Normalized Difference Vegetation Index (GNDVI) | Green biomass and Coverage; chlorophyll | (Gitelson et al., 1996; Gitelson and Merzlyak, 1998) |
| Greennees Index (GI) | Chlorophyll a, b | (Zarco-Tejada et al., 2005) |
| Leaf Chlorophyll Index (LCI) | Chlorophyll | (Pu et al., 2008) |
| Lichtenthaler Index (LIC) | Short term heat stress | (Lichtenthaler et al., 1996) |
| MERIS Terrestrial Chlorophyll Index (MTCI) | Chlorophyll | (Dash et al., 2010) |
| Modified Anthocyanin Reflectance Index (mARI) | Anthocyanins | (Gitelson et al., 2009) |
| Modified Chlorophyll Absorption Ratio Index (MCARI2) | Green leaf area index | (Haboudane et al., 2004) |
| Modified Chlorophyll Absorption in Reflectance Index (MCARI) | Chlorophyll | (Daughtry et al., 2000; Haboudane et al., 2004) |
| Modified Green Red Vegetation Index (MGRVI) | Biomass | (Bendig et al., 2015a) |
| Modified NDVI for Hyperspectral (mNDVI673) | Biomass | (Bargain et al., 2012) |
| Modified Normalized Difference at 705 (mND705) | Chlorophyll | (Sims and Gamon, 2002) |
| Modified Simple Ratio at 705 (mSR705) | Chlorophyll | (Sims and Gamon, 2002) |
| Modified Soil Adjusted Vegetation Index (MSAVI) | Minimize soil background influences, LAI, chlorophyll | (Qi et al., 1994) |
| Normalized Area Vegetation Index (NAVI) | Chlorophyll | (Carmona et al., 2015) |
| Normalized Difference Spectral Index (NDSI) | Nitrogen nutrition index | (Zhao et al., 2018) |
| Normalized Difference Vegetation Index (NDVI) | Green Biomass and Coverage | (Rouse, 1973) |
| Normalized Green Red Difference Index (NGRDI) | Biomass, leaf water content, chlorophyll | (Tucker, 1979) |
| Normalized Total Pigment Chlorophyll a Ratio (NPCI) | Chlorophyll-a | (Peñuelas et al., 1993) |
| Optimized Soil-Adjusted Vegetation Index (OSAVI) | Minimize soil background influences, LAI, chlorophyll | (Rondeaux et al., 1996) |
| Plant Biochemical Index (PBI) | Dry weight, ground cover | (Rao et al., 2008) |
| Ratio Vegetation Index (RVI) | Monitoring vegetation cover | (Pearson and Miller, 1972) |
| Red Edge Chlorophyll Index (CIrededge) | Canopy chlorophyll | (Clevers and Kooistra, 2012) |
| Red Edge Chlorophyll Reflectance Index (RIrededge) | Chlorophyll | (Gitelson and Merzlyak, 2004; Costa et al., 2021) |
| Red Edge Normalized Difference Vegetation Index (reNDVI) | Chlorophyll | (Gitelson and Merzlyak, 1994) |
| Red Edge Ratio (SR478) | Rededge | (Sims and Gamon, 2002; Costa et al., 2021) |
| Red Green Blue Vegetation Index (RGBVI) | Biomass | (Bendig et al., 2015b) |
| Renormalized Difference Vegetation Index (RDVI) | Fpar | (Roujean and Breon, 1995) |
| Simple Ratio (SR) | Leaf area index, chlorophyll a | (Jordan, 1969) |
| Simple Ratio at 445 (SR445) | Anthocyanins | (Thorhaug et al., 2015) |
| Simple Ratio at 700 (SR700) | Chlorophyll | (Chappelle et al., 1992) |
| Simple Ratio at 705 (SR705) | Chlorophyll | (Gitelson and Merzlyak, 1994) |
| Soil Adjusted Vegetation Index(L=-0.2) (SAVI2) | Aboveground living biomass | (Ren et al., 2018) |
| Soil Adjusted vegetation Index(L=0.5) (SAVI) | Eliminated soil-induced variations in vegetation indices, LAI, chlorophyll | (Huete, 1988) |
| Soil cover | Soil cover | (Bunnik, 1981) |
| Structure Independent Pigment Index (SIPI) | Chlorophyll-a, carotenoid | (Penuelas et al., 1995; Costa et al., 2021) |
| TCARI/OSAVI | Chlorophyll | (Haboudane et al., 2002) |
| Triangular Greenness Index (TGI) | Chlorophyll, Leaf area index | (Hunt Jr. et al., 2011) |
| Triangular Vegetation Index (TVI) | Green leaf area index, chlorophyll | (Broge and Leblanc, 2001) |
| Vegetative Index (VEG) | Vegetation coverage | (Hague et al., 2006) |
| Visible Atmospherically Resistant Index (VARI) | Vegetation fraction | (Gitelson et al., 2002) |
| Wide Dynamic Range Vegetation Index (WDRVI) | Vegetation fraction | (Gitelson, 2004) |

Note: *B, G, R, RE, and NIR represent the reflectance for the blue, green, red, red-edge and near-infrared bands, respectively.*

# Supplementary Table 3. List of vegetation indices that showed a number of times significant correlations across three years of study.

| **Vegetation index** | **No. of times VIs show r ≥0.7** | | |
| --- | --- | --- | --- |
|  | **2020** | **2021** | **2022** |
| Anthocyanin Reflectance Index (ARI) | - | - | 4 |
| Green Chlorophyll Index (CIgreen) | - | 5 | 6 |
| Red Edge Chlorophyll Index (CIrededge) | - | - | 6 |
| Carotenoids Reflectance Index (CRIrededge) | - | - | 4 |
| Chlorophyll Vegetation Index (CVI) | - | 7 | - |
| Enhanced Vegetation Index (EVI) | 2 | - | 5 |
| Green Normalized Difference Vegetation Index (GNDVI) | - | - | 6 |
| Leaf Chlorophyll Index (LCI) | 4 | 7 | 5 |
| Modified Anthocyanin Reflectance Index (mARI) | - | - | 4 |
| Modified Chlorophyll Absorption Ratio Index (MCARI2) | 2 | - | 6 |
| Modified Normalized Difference at 705 (mND705) | 3 | 6 | 6 |
| Modified Soil Adjusted Vegetation Index (MSAVI) | 2 | - | 6 |
| Modified Simple Ratio at 705 (mSR705) | 3 | 6 | 6 |
| MERIS Terrestrial Chlorophyll Index (MTCI) | 4 | 7 | 5 |
| Normalized Area Vegetation Index (NAVI) | - | - | 6 |
| Normalized Difference Vegetation Index (NDVI) | - | - | 6 |
| Optimized Soil-Adjusted Vegetation Index (OSAVI) | 2 | - | 6 |
| Plant Biochemical Index (PBI) | - | 5 | 6 |
| Renormalized Difference Vegetation Index (RDVI) | 2 | - | 6 |
| Red Edge Normalized Difference Vegetation Index (reNDVI) | - | 6 | 6 |
| Green Chlorophyll Reflectance Index (RIgreen) | 2 | 6 | - |
| Red Edge Chlorophyll Reflectance Index (RIrededge) | 3 | 6 | 6 |
| Ratio Vegetation Index (RVI) | - | - | 6 |
| Soil Adjusted vegetation Index(L=0.5) (SAVI) | 2 | - | 6 |
| Simple Ratio (SR) | - | - | 6 |
| Simple Ratio at 445 (SR445) | - | - | 6 |
| Simple Ratio at 705 (SR705) | - | 6 | 6 |
| Tcari/Osavi | - | - | 4 |
| Triangular Greenness Index (TGI) | - | 5 | - |
| Triangular Vegetation Index (TVI) | - | - | 4 |
| Wide Dynamic Range Vegetation Index (WDRVI) | - | - | 6 |

# Supplementary Table 4. Association between stable VIs and corn yield influenced by cover crop collected at R1 stages and corn yield under rainfed environments.

| **Vegetation Index** | **2020** | | | **2021** | | | **2022** | | |
| --- | --- | --- | --- | --- | --- | --- | --- | --- | --- |
|  | **Cover crop** | **R^2^** | **MAPE** | **Cover crop** | **R^2^** | **MAPE** | **Cover crop** | **R^2^** | **MAPE** |
| **LCI** | NCC | 0.54ns | 0.53 | NCC | 0.97* | 2.2 | NCC | 0.92* | 2.16 |
|  | Peas | 0.41ns | 3.69 | Peas | 0.89*** | 4.09 | Peas | 0.74** | 4.42 |
|  | Radish | 0.81** | 2.1 | Radish | 0.53* | 3.89 | Mixed | 0.59* | 7.16 |
|  | Rye | 0.45ns | 2.2 | Rye | 0.93*** | 1.26 | Rye | 0.43ns | 7.06 |
| **mND705** | NCC | 0.49ns | 0.55 | NCC | 0.97* | 1.71 | NCC | 0.9ns | 2.25 |
|  | Peas | 0.4ns | 3.79 | Peas | 0.83** | 4.9 | Peas | 0.67* | 4.99 |
|  | Radish | 0.85** | 1.8 | Radish | 0.3ns | 4.61 | Mixed | 0.5ns | 7.73 |
|  | Rye | 0.43ns | 2.25 | Rye | 0.82** | 1.89 | Rye | 0.47ns | 6.94 |
| **mSR705** | NCC | 0.49ns | 0.55 | NCC | 0.97* | 1.86 | NCC | 0.87ns | 2.67 |
|  | Peas | 0.38ns | 3.85 | Peas | 0.85** | 4.54 | Peas | 0.65* | 5.09 |
|  | Radish | 0.85** | 1.83 | Radish | 0.31ns | 4.63 | Mixed | 0.49ns | 7.68 |
|  | Rye | 0.43ns | 2.25 | Rye | 0.82** | 1.86 | Rye | 0.44ns | 7.18 |
| **MTCI** | NCC | 0.53ns | 0.54 | NCC | 0.98* | 1.7 | NCC | 0.9ns | 2.59 |
|  | Peas | 0.39ns | 3.75 | Peas | 0.9*** | 3.69 | Peas | 0.71** | 4.57 |
|  | Radish | 0.81** | 2.03 | Radish | 0.54* | 3.83 | Mixed | 0.58* | 7.15 |
|  | Rye | 0.45ns | 2.21 | Rye | 0.92*** | 1.31 | Rye | 0.4ns | 7.3 |
| **RIrededge** | NCC | 0.49ns | 0.55 | NCC | 0.97* | 1.86 | NCC | 0.87ns | 2.67 |
|  | Peas | 0.38ns | 3.85 | Peas | 0.85** | 4.54 | Peas | 0.65* | 5.09 |
|  | Radish | 0.85** | 1.83 | Radish | 0.31ns | 4.63 | Mixed | 0.49ns | 7.68 |
|  | Rye | 0.43ns | 2.25 | Rye | 0.82** | 1.86 | Rye | 0.44ns | 7.18 |

Note: Leaf Chlorophyll Index (LCI), MERIS Terrestrial Chlorophyll Index (MTCI), Red Edge Chlorophyll Reflectance Index (RIrededge), Modified Simple Ratio at 705 (mSR705) and Modified Normalized Difference at 705 (mND705). *, ** and *** indicate the regression is significant at *p*<0.05, *p*<0.01 and *p*<0.001 respectively. ‘ns’ indicates non-significant. MAPE: mean absolute percentage error.

**References associated with Supplementary Table 2.**

Bargain, A., Robin, M., Le Men, E., Huete, A., and Barillé, L. (2012). Spectral response of the seagrass Zostera noltii with different sediment backgrounds. *Aquatic Botany* 98, 45–56. doi: 10.1016/j.aquabot.2011.12.009.

Bendig, J., Yu, K., Aasen, H., Bolten, A., Bennertz, S., Broscheit, J., et al. (2015a). Combining UAV-based plant height from crop surface models, visible, and near infrared vegetation indices for biomass monitoring in barley. *International Journal of Applied Earth Observation and Geoinformation* 39, 79–87. doi: 10.1016/j.jag.2015.02.012.

Bendig, J., Yu, K., Aasen, H., Bolten, A., Bennertz, S., Broscheit, J., et al. (2015b). Combining UAV-based plant height from crop surface models, visible, and near infrared vegetation indices for biomass monitoring in barley. *International Journal of Applied Earth Observation and Geoinformation* 39, 79–87. doi: 10.1016/j.jag.2015.02.012.

Broge, N. H., and Leblanc, E. (2001). Comparing prediction power and stability of broadband and hyperspectral vegetation indices for estimation of green leaf area index and canopy chlorophyll density. *Remote Sensing of Environment* 76, 156–172. doi: 10.1016/S0034-4257(00)00197-8.

Bunnik, N. J. J. (1981). Spectral reflectance characteristics of agricultural crops and application to crop growth monitoring. *Advances in Space Research* 1, 21–40. doi: 10.1016/0273-1177(81)90379-3.

Carmona, F., Rivas, R., and Fonnegra, D. C. (2015). Vegetation Index to estimate chlorophyll content from multispectral remote sensing data. *European Journal of Remote Sensing* 48, 319–326. doi: 10.5721/EuJRS20154818.

Chappelle, E. W., Kim, M. S., and McMurtrey, J. E. (1992). Ratio analysis of reflectance spectra (RARS): An algorithm for the remote estimation of the concentrations of chlorophyll A, chlorophyll B, and carotenoids in soybean leaves. *Remote Sensing of Environment* 39, 239–247. doi: 10.1016/0034-4257(92)90089-3.

Clevers, J. G. P. W., and Kooistra, L. (2012). Using Hyperspectral Remote Sensing Data for Retrieving Canopy Chlorophyll and Nitrogen Content. *IEEE Journal of Selected Topics in Applied Earth Observations and Remote Sensing* 5, 574–583. doi: 10.1109/JSTARS.2011.2176468.

Costa, V., Serôdio, J., Lillebø, A. I., and Sousa, A. I. (2021). Use of hyperspectral reflectance to non-destructively estimate seagrass Zostera noltei biomass. *Ecological Indicators* 121, 107018. doi: 10.1016/j.ecolind.2020.107018.

Dash, J., Curran, P. J., Tallis, M. J., Llewellyn, G. M., Taylor, G., and Snoeij, P. (2010). Validating the MERIS Terrestrial Chlorophyll Index (MTCI) with ground chlorophyll content data at MERIS spatial resolution. *International Journal of Remote Sensing* 31, 5513–5532. doi: 10.1080/01431160903376340.

Datt, B. (1998). Remote Sensing of Chlorophyll a, Chlorophyll b, Chlorophyll a+b, and Total Carotenoid Content in Eucalyptus Leaves. *Remote Sensing of Environment* 66, 111–121. doi: 10.1016/S0034-4257(98)00046-7.

Daughtry, C. S. T., Walthall, C. L., Kim, M. S., de Colstoun, E. B., and McMurtrey, J. E. (2000). Estimating Corn Leaf Chlorophyll Concentration from Leaf and Canopy Reflectance. *Remote Sensing of Environment* 74, 229–239. doi: 10.1016/S0034-4257(00)00113-9.

Fyfe, S. K. (2003). Spatial and temporal variation in spectral reflectance: Are seagrass species spectrally distinct? *Limnology and Oceanography* 48, 464–479. doi: 10.4319/lo.2003.48.1_part_2.0464.

Gitelson, A. A. (2004). Wide Dynamic Range Vegetation Index for Remote Quantification of Biophysical Characteristics of Vegetation. *Journal of Plant Physiology* 161, 165–173. doi: 10.1078/0176-1617-01176.

Gitelson, A. A., Chivkunova, O. B., and Merzlyak, M. N. (2009). Nondestructive estimation of anthocyanins and chlorophylls in anthocyanic leaves. *American Journal of Botany* 96, 1861–1868. doi: 10.3732/ajb.0800395.

Gitelson, A. A., Kaufman, Y. J., and Merzlyak, M. N. (1996). Use of a green channel in remote sensing of global vegetation from EOS-MODIS. *Remote Sensing of Environment* 58, 289–298. doi: 10.1016/S0034-4257(96)00072-7.

Gitelson, A. A., Kaufman, Y. J., Stark, R., and Rundquist, D. (2002). Novel algorithms for remote estimation of vegetation fraction. *Remote Sensing of Environment* 80, 76–87. doi: 10.1016/S0034-4257(01)00289-9.

Gitelson, A. A., and Merzlyak, M. N. (1998). Remote sensing of chlorophyll concentration in higher plant leaves. *Advances in Space Research* 22, 689–692. doi: 10.1016/S0273-1177(97)01133-2.

Gitelson, A. A., Viña, A., Ciganda, V., Rundquist, D. C., and Arkebauer, T. J. (2005). Remote estimation of canopy chlorophyll content in crops. *Geophysical Research Letters* 32. doi: 10.1029/2005GL022688.

Gitelson, A., and Merzlyak, M. (2004). Non-Destructive Assessment of Chlorophyll Carotenoid and Anthocyanin Content in Higher Plant Leaves: Principles and Algorithms. *Remote Sensing for Agriculture and the Environment*.

Gitelson, A., and Merzlyak, M. N. (1994). Spectral Reflectance Changes Associated with Autumn Senescence of Aesculus hippocastanum L. and Acer platanoides L. Leaves. Spectral Features and Relation to Chlorophyll Estimation. *Journal of Plant Physiology* 143, 286–292. doi: 10.1016/S0176-1617(11)81633-0.

Haboudane, D., Miller, J. R., Pattey, E., Zarco-Tejada, P. J., and Strachan, I. B. (2004). Hyperspectral vegetation indices and novel algorithms for predicting green LAI of crop canopies: Modeling and validation in the context of precision agriculture. *Remote Sensing of Environment* 90, 337–352. doi: 10.1016/j.rse.2003.12.013.

Haboudane, D., Miller, J. R., Tremblay, N., Zarco-Tejada, P. J., and Dextraze, L. (2002). Integrated narrow-band vegetation indices for prediction of crop chlorophyll content for application to precision agriculture. *Remote Sensing of Environment* 81, 416–426. doi: 10.1016/S0034-4257(02)00018-4.

Hague, T., Tillett, N. D., and Wheeler, H. (2006). Automated Crop and Weed Monitoring in Widely Spaced Cereals. *Precision Agric* 7, 21–32. doi: 10.1007/s11119-005-6787-1.

Huete, A., Didan, K., Miura, T., Rodriguez, E. P., Gao, X., and Ferreira, L. G. (2002). Overview of the radiometric and biophysical performance of the MODIS vegetation indices. *Remote Sensing of Environment* 83, 195–213. doi: 10.1016/S0034-4257(02)00096-2.

Huete, A. R. (1988). A soil-adjusted vegetation index (SAVI). *Remote Sensing of Environment* 25, 295–309. doi: 10.1016/0034-4257(88)90106-X.

Hunt Jr., E. R., Daughtry, C. S. T., Eitel, J. U. H., and Long, D. S. (2011). Remote Sensing Leaf Chlorophyll Content Using a Visible Band Index. *Agronomy Journal* 103, 1090–1099. doi: 10.2134/agronj2010.0395.

Jordan, C. F. (1969). Derivation of Leaf-Area Index from Quality of Light on the Forest Floor. *Ecology* 50, 663–666. doi: 10.2307/1936256.

Karcher, D. E., and Richardson, M. D. (2003). Quantifying Turfgrass Color Using Digital Image Analysis. *Crop Science* 43, 943–951. doi: 10.2135/cropsci2003.9430.

Lichtenthaler, H. K., Lang, M., Sowinska, M., Heisel, F., and Miehé, J. A. (1996). Detection of Vegetation Stress Via a New High Resolution Fluorescence Imaging System. *Journal of Plant Physiology* 148, 599–612. doi: 10.1016/S0176-1617(96)80081-2.

Louhaichi, M., Borman, M. M., and Johnson, D. E. (2001). Spatially Located Platform and Aerial Photography for Documentation of Grazing Impacts on Wheat. *Geocarto International* 16, 65–70. doi: 10.1080/10106040108542184.

Pearson, R. L., and Miller, L. D. (1972). Remote mapping of standing crop biomass for estimation of the productivity of the short-grass prairie, Pawnee National Grasslands, Colorado. in *Proceedings of the Eight International Symposium on Remote Sensing of Environment, 2–6 October 1972* (Ann Arbor, Michigan USA: Willow Run Laboratories, Environmental Research Institute of Michigan), 1355–1379.

Penuelas, J., Frederic, B., and Filella, I. (1995). Semi-Empirical Indices to Assess Carotenoids/Chlorophyll-a Ratio from Leaf Spectral Reflectance. *Photosynthetica* 31, 221–230.

Peñuelas, J., Gamon, J. A., Griffin, K. L., and Field, C. B. (1993). Assessing community type, plant biomass, pigment composition, and photosynthetic efficiency of aquatic vegetation from spectral reflectance. *Remote Sensing of Environment* 46, 110–118. doi: 10.1016/0034-4257(93)90088-F.

Prabhakara, K., Hively, W. D., and McCarty, G. W. (2015). Evaluating the relationship between biomass, percent groundcover and remote sensing indices across six winter cover crop fields in Maryland, United States. *International Journal of Applied Earth Observation and Geoinformation* 39, 88–102. doi: 10.1016/j.jag.2015.03.002.

Pu, R., Gong, P., and Yu, Q. (2008). Comparative Analysis of EO-1 ALI and Hyperion, and Landsat ETM+ Data for Mapping Forest Crown Closure and Leaf Area Index. *Sensors* 8, 3744–3766. doi: 10.3390/s8063744.

Qi, J., Chehbouni, A., Huete, A. R., Kerr, Y. H., and Sorooshian, S. (1994). A modified soil adjusted vegetation index. *Remote Sensing of Environment* 48, 119–126. doi: 10.1016/0034-4257(94)90134-1.

Rao, N. R., Garg, P. K., Ghosh, S. K., and Dadhwal, V. K. (2008). Estimation of leaf total chlorophyll and nitrogen concentrations using hyperspectral satellite imagery. *The Journal of Agricultural Science* 146, 65–75. doi: 10.1017/S0021859607007514.

Ren, H., Zhou, G., and Zhang, F. (2018). Using negative soil adjustment factor in soil-adjusted vegetation index (SAVI) for aboveground living biomass estimation in arid grasslands. *Remote Sensing of Environment* 209, 439–445. doi: 10.1016/j.rse.2018.02.068.

Rondeaux, G., Steven, M., and Baret, F. (1996). Optimization of soil-adjusted vegetation indices. *Remote Sensing of Environment* 55, 95–107. doi: 10.1016/0034-4257(95)00186-7.

Rorie, R. L., Purcell, L. C., Mozaffari, M., Karcher, D. E., King, C. A., Marsh, M. C., et al. (2011). Association of “Greenness” in Corn with Yield and Leaf Nitrogen Concentration. *Agronomy Journal* 103, 529–535. doi: 10.2134/agronj2010.0296.

Roujean, J.-L., and Breon, F.-M. (1995). Estimating PAR absorbed by vegetation from bidirectional reflectance measurements. *Remote Sensing of Environment* 51, 375–384. doi: 10.1016/0034-4257(94)00114-3.

Rouse, J. W. (1973). Monitoring the vernal advancement and retrogradation (green wave effect) of natural vegetation. Available at: https://ntrs.nasa.gov/citations/19730016613 [Accessed April 8, 2022].

Sims, D. A., and Gamon, J. A. (2002). Relationships between leaf pigment content and spectral reflectance across a wide range of species, leaf structures and developmental stages. *Remote Sensing of Environment* 81, 337–354. doi: 10.1016/S0034-4257(02)00010-X.

Thorhaug, A., Berlyn, G. P., Poulos, H. M., and Goodale, U. M. (2015). Pollutant tracking for 3 Western North Atlantic sea grasses by remote sensing: Preliminary diminishing white light responses of Thalassia testudinum, Halodule wrightii, and Zostera marina. *Marine Pollution Bulletin* 97, 460–469. doi: 10.1016/j.marpolbul.2015.05.028.

Tucker, C. J. (1979). Red and photographic infrared linear combinations for monitoring vegetation. *Remote Sensing of Environment* 8, 127–150. doi: 10.1016/0034-4257(79)90013-0.

Vincini, M., Frazzi, E., and D’Alessio, P. (2008). A broad-band leaf chlorophyll vegetation index at the canopy scale. *Precision Agric* 9, 303–319. doi: 10.1007/s11119-008-9075-z.

Zarco-Tejada, P. J., Berjón, A., López-Lozano, R., Miller, J. R., Martín, P., Cachorro, V., et al. (2005). Assessing vineyard condition with hyperspectral indices: Leaf and canopy reflectance simulation in a row-structured discontinuous canopy. *Remote Sensing of Environment* 99, 271–287. doi: 10.1016/j.rse.2005.09.002.

Zhao, B., Duan, A., Ata-Ul-Karim, S. T., Liu, Z., Chen, Z., Gong, Z., et al. (2018). Exploring new spectral bands and vegetation indices for estimating nitrogen nutrition index of summer maize. *European Journal of Agronomy* 93, 113–125. doi: 10.1016/j.eja.2017.12.006.
